# The complete mitochondrial genome of *Hypomesus olidus* (Osmeriformes: Osmeridae)

**DOI:** 10.3109/19401736.2015.1007313

**Published:** 2017-10-19

**Authors:** Xiaohui Bai, Zhang Luo, Shouming Feng, Zhijing Sun, Hui-Min Wu, Chun-Yan Li, Ju-Feng Jiang, Xiaolian Liu, Na Wang

**Affiliations:** Aquaculture Technology Research Laboratory, Tianjin Fisheries Research Institute, Tianjin, People’s Republic of China

**Keywords:** *Hypomesus olidus*, mitochondrial genome, Osmeridae

## Abstract

Pond smelt *Hypomesus olidus* is a small-sized economic species of freshwater fish with delicious meat and high output. In this study, the complete mitochondrial genome of *H. olidus* sequenced to be 16,786 bp in length, including 13 protein-coding genes, 2 ribosomal RNAs, 22 transfer RNAs and a control region. The overall base composition of *H. olidus* is 24.3% for A, 27.3% for T, 29.2% for C and 19.2% for G, with a slight A + T bias. The mitogenome sequence data may provide useful information to the population genetics analysis of *H. olidus* and the elucidation of evolutionary mechanisms in *Hypomesus* fish.

Pond smelt *Hypomesus olidus*, a small-sized economic species of freshwater fish, is one of the five species comprising the genus *Hypomesus*, family Osmeridae. *Hypomesus olidus* is distributed in northwest and northeast Pacific as well as along the North American and Arctic coastlines (Ilves and Taylor 2008; Skurikhina et al. 2012). In China, it is found in the Amur River, Tumen River, the Yalu River and the Dayanghe River in the Liaodong Peninsula, etc. (Du et al. 2001). For delicious meat and high output, it has been introduced in many domestic lakes and reservoirs in recent years. Phylogenetic reconstruction based on the analysis of morphological characters and partial mitochondrial and nuclear genes revealed that *Hypomesus* has a complicated taxonomic history (Saruwatari et al. 1997; Sidorov and Pichugin 2004; Ilves and Taylor 2007; Skurikhina et al. 2012). In this study, the complete mitochondrial genome of *H. olidus* was sequenced with the aim of obtaining genetic information essential to inferring its evolution and phylogenetic analysis of *Hypomesus* fish.

*Hypomesus olidus* were collected from Yuqiao Reservoir, Tianjin Municipality in China. Total genomic DNA from muscle tissue was extracted using the DNeasy Tissue Kit (Qiagen, Valencia, CA). Eleven amplification primers were designed to amplify the complete mitogenome based on *Hypomesus nipponensis*’s mitochondrial nucleotide sequences (GenBank accession no. HM106489.1). The PCR products were purified and inserted into the pMD18T vector. Then, the recombinant plasmids were transformed into *Escherichia coli* strain TOP10 competent cells and sequenced by Sangon Biotech (Beijing, China). Sequences were assembled, and the mitogenome sequence of *H. olidus* has been deposited in the GenBank with accession number of KP281293.

The mitogenome length of *H. olidus* is 16,786 bp and contains 22 tRNA genes, 13 protein-coding genes (PCGs), 2 rRNA genes and a displacement loop region (D-loop) (Table 1). The overall base composition of *H. olidus* is 24.3% for A, 27.3% for T, 29.2% for C and 19.2% for G. The A + T content is higher than G + C content as was found in many other fishes (Wang et al. 2011; Huang et al. 2015). Most of the mitochondrial genes of this species are encoded on heavy strand except for *ND6* and eight tRNA genes (*Gln*, *Ala*, *Asn*, *Cys*, *Tyr*, *Ser*, *Glu* and *Pro*), which are encoded on light strand. A total of four overlaps and 10 intergenic spacer regions among PCGs and tRNAs were identified in this genome (Table 1). All of the 13 PCGs contain the orthodox start codon ATG except *COX1* (GTG) and *ND6* (CTA). However, the end codons of the 13 PCGs are varied with TAA, TA or T. Six PCGs used TAA (*ND1*, *COI*, *ATPase8*, *ATPase6*, *ND4L* and *ND5*) as the stop codon. The rest PCGs ended with the incomplete stop codon TA (*COX3*) or T (*ND2*, *COX2*, *ND3*, *ND4*, *ND6* and *Cytb*) as the stop codons. The control region (D-loop) is located between the *tRNA*-*Pro* and *tRNA*-*Phe* genes, and its length is 1103 bp. The mitogenomes of *H. olidus* and its congeneric species *H. nipponensis* have 99% nucleotide sequence identity (Li et al. 2010).

**Table 1.** Characteristics of the mitochondrial genome of *H. olidus*.

	Location		Size of nucleotide (bp)		Codon		Intergenic nucleotide^a^	
Locus	Start	Stop		Amino acid	Start	Stop		Strand^b^
*tRNA^*Phe*^*	1	68	68					H
*12SrRNA*	69	1013	945				0	H
*tRNA^*Val*^*	1014	1084	71				0	H
*16SrRNA*	1085	2790	1706				0	H
*tRNA^*Leu(UUR)*^*	2797	2870	74				0	H
*ND1*	2871	3845	975	324	ATG	TAA	0	H
*tRNA ^*Ile*^*	3849	3920	72				3	H
*tRNA^*Gln*^*	3921	3990	70				0	L
*tRNA^*Met*^*	3990	4058	69				–1	H
*ND2*	4063	5113	1051	350	ATG	T– –	0	H
*tRNA^*Trp*^*	5114	5185	72				0	H
*tRNA^*Ala*^*	5187	5255	69				1	L
*tRNA^*Asn*^*	5257	5329	73				1	L
*tRNA^*Cys*^*	5357	5421	65				27	L
*tRNA ^*Tyr*^*	5421	5488	68				1	L
*COX1*	5491	7041	1551	516	GTG	TAA	2	H
*tRNA^*Ser(UCN)*^*	7042	7112	71				0	L
*tRNA^*Asp*^*	7117	7189	73				4	H
*COX2*	7204	7894	691	230	ATG	T– –	14	H
*tRNA^*Lys*^*	7895	7969	75				0	H
*ATP8*	7971	8138	168	55	ATG	TAA	1	H
*ATP6*	8129	8812	684	227	ATG	TAA	–10	H
*COX3*	8812	9596	785	261	ATG	TA–	0	H
*tRNA^*Gly*^*	9597	9667	71				0	H
*ND3*	9668	10,016	349	116	ATG	T––	0	H
*tRNA^*Arg*^*	10,017	10,086	70				0	H
*ND4L*	10,087	10,383	297	98	ATG	TAA	0	H
*ND4*	10,377	11,757	1381	460	ATG	T––	–7	H
*tRNA^*His*^*	11,758	11,826	69				0	H
*tRNA^*Ser(AGY)*^*	11,827	11,895	69				0	H
*tRNA^*Leu(CUN)*^*	11,896	11,969	74				0	H
*ND5*	11,970	13,808	1839	612	ATG	TAA	0	H
*ND6*	13,805	14,326	522	173	CTA	T––	–4	L
*tRNA^*Glu*^*	14,327	14,396	70				0	L
*Cytb*	14,401	15,541	1141	380	ATG	T––	4	H
*tRNA^*Thr*^*	15,542	15,613	72				0	H
*tRNA^*Pro*^*	15,614	15,683	70				0	L
D-loop	15,684	16,786	1103				0	H

^a^ Numbers correspond to the nucleotides separating different genes, and negative numbers represent the overlapping nucleotides.

^b^ H and L indicate heavy and light strands, repectively.
